# DNA–melamine hybrid molecules: from self-assembly to nanostructures

**DOI:** 10.3762/bjnano.6.148

**Published:** 2015-06-30

**Authors:** Rina Kumari, Shib Shankar Banerjee, Anil K Bhowmick, Prolay Das

**Affiliations:** 1 Department of Chemistry, Indian Institute of Technology Patna, Patna 800013, India; 2 Department of Materials Science and Engineering, Indian Institute of Technology Patna, Patna 800013, India,; 3 Rubber Technology Centre, Indian Institute of Technology Kharagpur, Kharagpur 721302, India

**Keywords:** DNA–organic hybrid, melamine, nanostructures, phosphoramidation, self-assembly

## Abstract

Single-stranded DNA–melamine hybrid molecular building blocks were synthesized using a phosphoramidation cross-coupling reaction with a zero linker approach. The self-assembly of the DNA–organic hybrid molecules was achieved by DNA hybridization. Following self-assembly, two distinct types of nanostructures in the form of linear chains and network arrays were observed. The morphology of the self-assembled nanostructures was found to depend on the number of DNA strands that were attached to a single melamine molecule.

## Findings

The importance of DNA in the field of nanotechnology stems from the fact that DNA is a macromolecule on the nanoscale with self-assembling properties [1–2]. The molecular recognition through base pairing in DNA makes it an attractive molecular scaffold for the precise positioning of different molecules on the nanoscale [3–4]. DNA has been used to create nanostructures through hybridization-mediated self-assembly for molecular electronics and sensing applications [5–7]. DNA–organic hybrid structures have demonstrated great potential to produce molecular-scale building blocks. The covalent conjugation of the nucleobases with small molecules has been found to be a useful method to improve the properties of their natural counterparts such as nucleobase pairing fidelity, duplex stability, and for creating complex structures [8–10]. This promising strategy of building DNA nanostructures involving DNA–organic hybrid building blocks has been used to create electrochemical DNA sensors and systems that displayed redox, photophysical, photochemical or catalytic activities [11–12].

While there are definite advantages of using DNA–organic hybrid structures over DNA-only based nanostructures, there are challenges regarding their solubility as well as reactivity issues. In most cases, a DNA synthesizer based on phosphoramidite chemistry is used to introduce chemical modifications in the oligonucleotides [13–15]. However, the directionality of the single-stranded DNA (ssDNA) sequences obtained through a DNA synthesizer is limited only to the terminal positions. This could limit further DNA extension in both directions. As a recent trend, DNA–organic molecule hybrid structures are being produced post-synthesis to add versatility to the building blocks that are subsequently used to assemble complex nanostructures [16–17]. The repertoire of organic molecules is practically infinite, and the possibilities for developing novel DNA−organic molecular hybrid structures are endless. The judicious selection of organic molecules can result in unique DNA-based nanostructures for application in molecular and cellular biophysics, as biomimetic systems, in energy transfer and photonics, and in diagnostics and therapeutics [18–21]. Moreover, as a bottom-up technique, such a methodology can contribute to molecular electronics where tunable electronic properties and templated metallization are frequently warranted [22–24].

**Scheme 1:**
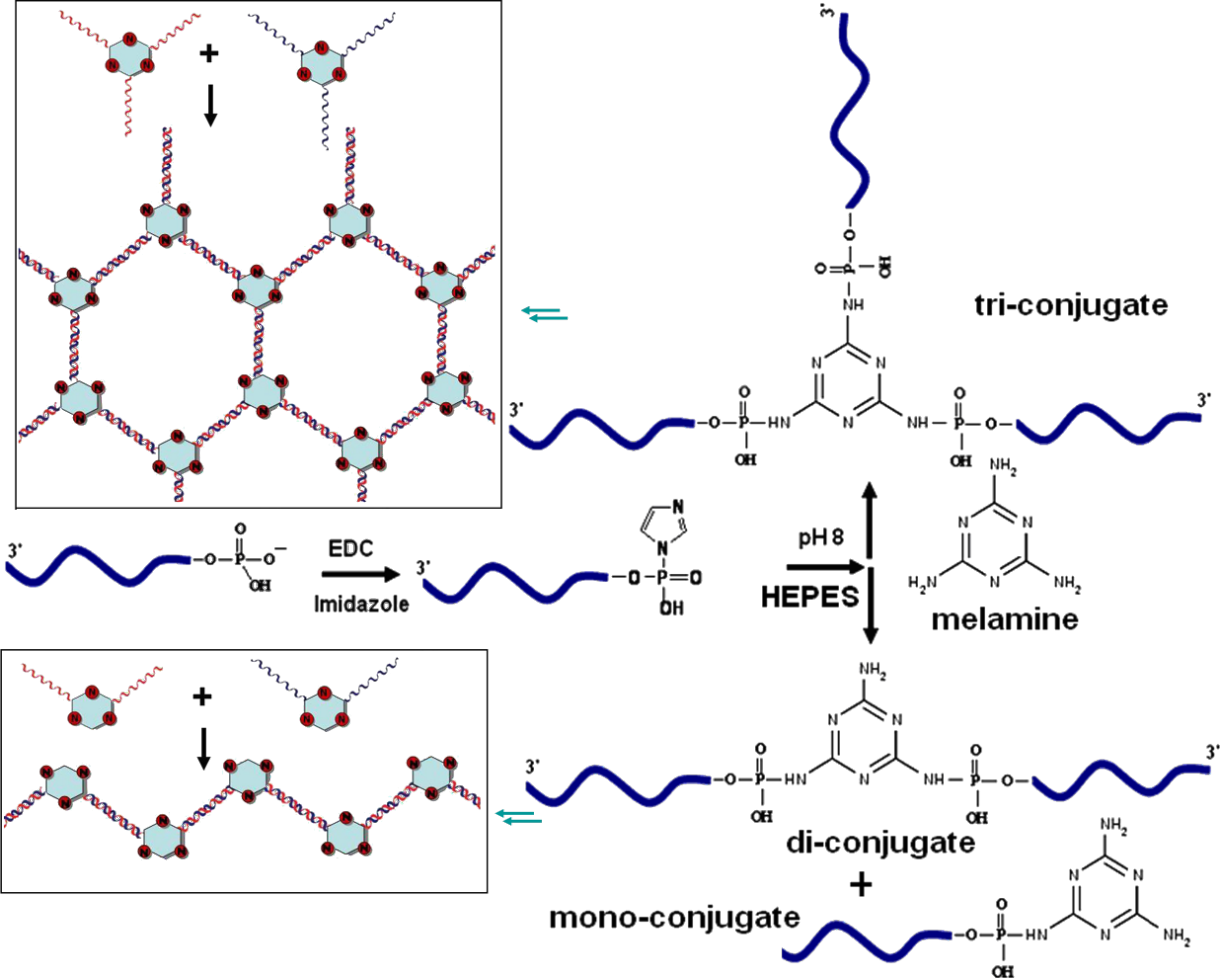
Synthesis of ssDNA–melamine conjugates by a phosphoramidation reaction.

Herein, we report the facile creation of DNA–organic hybrid molecules and demonstrate their self-assembly to create nanostructures. The organic molecule used here is melamine, a nitrogen-rich, planar, heterocyclic, aromatic compound, having multiple pendant amine functionality. Although, melamine has been previously used to generate nanostructures, it has not been explored to create self-assembled nanostructures exclusively with DNA [25–26]. The covalent conjugation of ssDNA with aromatic amine groups of melamine and the subsequent construction of two distinct types of nanoassemblies out of the DNA–organic hybrid molecules are demonstrated here for the first time (Scheme 1).

The 1-ethyl-3-(3-dimethylaminopropyl)carbodiimide (EDC) and imidazole-mediated phosphoramidation cross-coupling reaction between aliphatic primary amine moieties and a terminal phosphate group of DNA was previously reported [27–28]. We optimized the reaction for the coupling of the aromatic amines of melamine with the 5’-terminal phosphate groups of two self-complementary ssDNA molecules (24 bases long, Supporting Information File 1, Table S1) using a zero linker approach.

The terminal phosphomonoester groups of the oligomers, which is more nucleophilic for activation than the internucleotidic phosphodiesters of oligonucleotides, were activated by EDC in the presence of imidazole as a catalyst in aqueous solution. The 5’-phosphorimidazolide intermediate further reacts in situ with the primary aromatic amines of the melamine to give the desired product. In this study, an excess of ssDNA (10×) was used so that the probability of multiple DNA strands conjoined to a melamine molecule is increased. The solution-phase phosphoramidation cross coupling was confirmed by polyacrylamide gel electrophoresis (PAGE), reversed phase high performance liquid chromatography (RP-HPLC), matrix-assisted laser desorption/ionization (MALDI) and electrospray ionization (ESI) mass spectrometry (MS).

**Figure 1:**
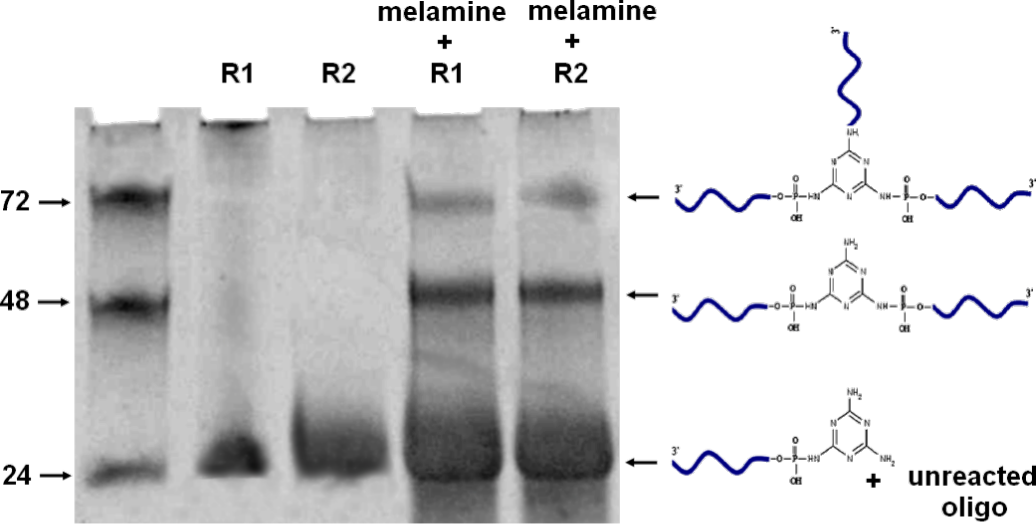
30% denaturing PAGE analysis of the reaction mixture of ssDNA and melamine.

The reaction products were purified by dialysis (*M*_W_ 1000 Da) to eliminate the small molecule impurities and tested using denaturing PAGE to distinguish between the covalent attachment and non-covalent association and to identify the reaction products (Figure 1). A clear separation of the bands for the di-branched and tri-branched ssDNA–melamine conjugates was observed. However, the unreacted DNA and singly conjugated DNA–melamine conjugate were merged as a single broad band at the bottom of the gel. The yield of the tri-branched ssDNA–melamine conjugate from the reaction was estimated as approximately 7–8% as compared to that of 25–28% for the di-branched counterpart. The poor yield of the former is attributed to steric hindrance offered by the DNA strands as well as the electronic effect that might decrease the nucleophilicity of the amine nitrogen. The yield was calculated by quantitative analysis of the band intensity from the PAGE images.

The confirmation of conjugation was also obtained from analytical RP-HPLC chromatograms where DNA–melamine conjugates show three distinct peaks corresponding to mono- (along with the unreacted oligomer), di- and tri-conjugates, respectively (see Supporting Information File 1, Figure S1). The retention times for the conjugates of the complementary oligomers are slightly different due to the difference in the DNA sequences (see Supporting Information File 1, Table S1). MALDI–TOF MS as well as ESIMS were performed on the PAGE-purified DNA–melamine conjugates (see Supporting Information File 1, Figures S2, S3). The mass spectra show the formation of di-branched and tri-branched DNA–melamine conjugate for both the ssDNA oligomers and unambiguously prove that the melamine is covalently conjugated to two or three ssDNA.

The di-branched DNA–melamine conjugate ((R1)_2_–melamine) was allowed to self-assemble with the corresponding di-branched hybrid with complementary DNA sequence ((R2)_2_–melamine) by annealing in sodium phosphate buffer at pH 7.0. This self-assembly through DNA hybridization is hypothesized to yield a linear chain of the DNA–melamine hybrid. Similarly, one of the tri-branched conjugates ((R1)_3_–melamine) was annealed with the corresponding tri-branched counterpart having the complementary DNA sequence ((R2)_3_–melamine) to produce a nanoassembly in the form of a network.

**Figure 2:**
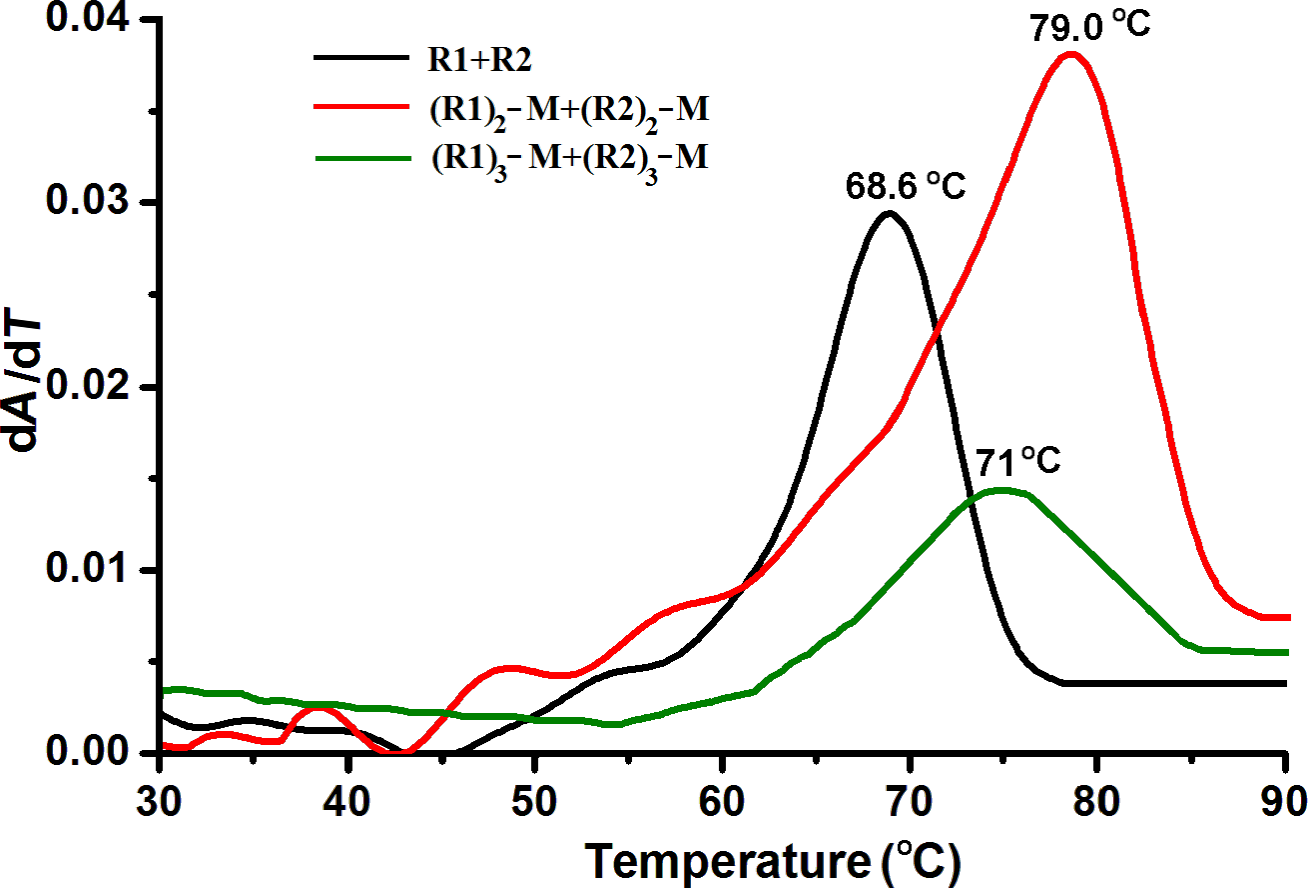
First-order derivatives of UV absorbance melting profiles at 260 nm for a control (R1–R2) and DNA–melamine hybrid self-assembly.

To investigate the outcome of the hybridization of the complementary DNA–melamine conjugates, thermal melting studies were performed with PAGE-purified hybrid conjugates (Figure 2). The assembly of the two complementary hybrid DNA species results in a substantial increase in the melting temperature in comparison to the unconjugated duplex DNA. The significant increase in the melting temperature for the self-assembled (R1)_2_–melamine with (R2)_2_–melamine of about 10 °C suggests the formation of a linear assembly upon annealing. As reported, an increase of the number of sticky-end association results in an increase in melting temperature, which is attributed to the additive effect [29–30]. In this case, a simple annealing of the complementary DNA of the covalent conjugates increases the melting temperature by ≈10 °C. The moderately sharp transition of the self-assembled di-branched conjugates also indicates the decreasing contribution of the terminal single-stranded dangling ends. This suggests the formation of a longer oligomer duplex with the hybrid monomers [15]. However, the assembly of the tri-branched conjugates, (R1)_3_–melamine with (R2)_3_–melamine, does not show a sharp melting temperature profile, indicating the absence of any sequential release of oligomers upon melting. In contrast to the assembly of di-branched conjugates, the increase in the melting temperature was only ≈3 °C. In general, the melting of a network proceeds faster than a corresponding concatemeric duplex, which was observed here [31–32]. The broadening of the differential peak of the tri-branched conjugates may be a result of several partially overlapped transitions due to end and junction destabilization, irregular duplex formation or the absence of DNA–DNA strand interaction [33–34]. These observations are indirect proof of the formation of two distinct types of higher order structures from the di-branched and tri-branched DNA–melamine conjugates. Overall, this implies that by using small and structurally well-defined organic molecules, it is possible to increase the stability and melting temperature of DNA duplexes in a self-assembly.

**Figure 3:**
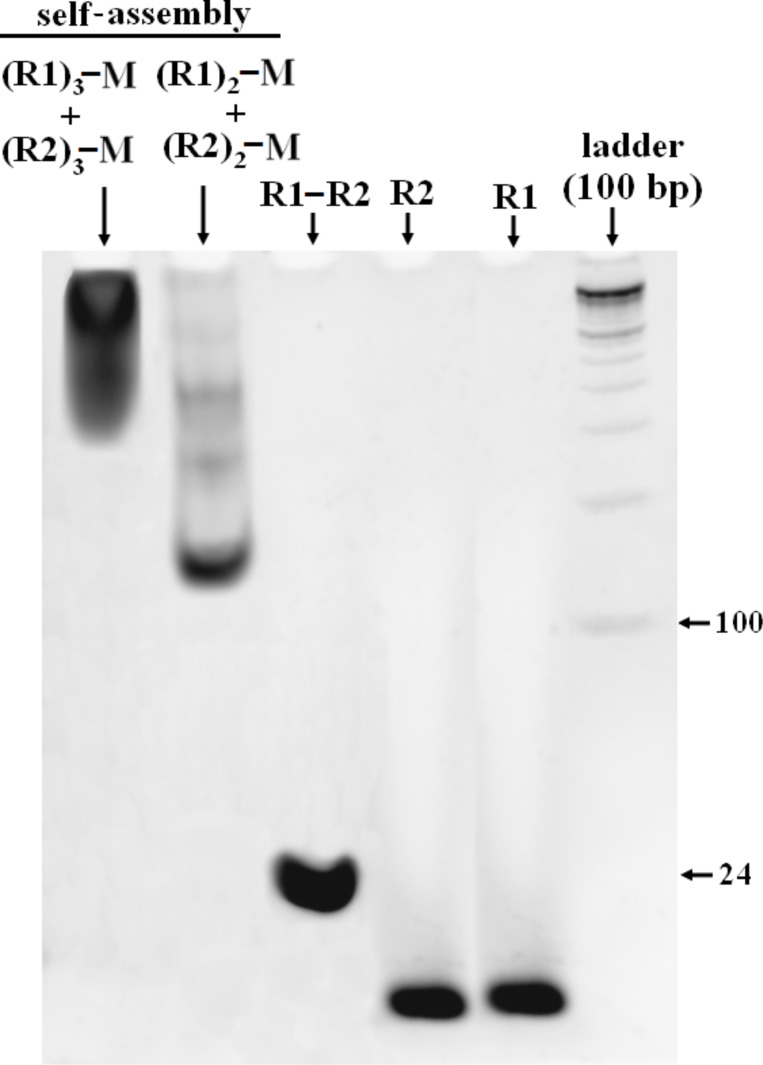
10% non-denaturing PAGE showing the difference in gel mobility of R1–R2 and the corresponding DNA–melamine conjugates in self-assembly, where M = melamine.

The self-assembly products were also characterized by native PAGE (Figure 3). Particularly noteworthy is the retardation in the mobility of the tri-conjugated assembly, which indicates the formation of higher-ordered structures. The smearing of the band of the tri-branched conjugate with native PAGE is due to a wide range of product distribution. The continuation of the tri-branched conjugate band below 300 bp (with respect to the ladder) indicates that few network arrays may be smaller than the linear counterpart in terms of DNA content. The assembly of di-branched conjugates moves in the gel, albeit with a much slower gel mobility than the normal R1–R2 oligomer duplex.

**Figure 4:**
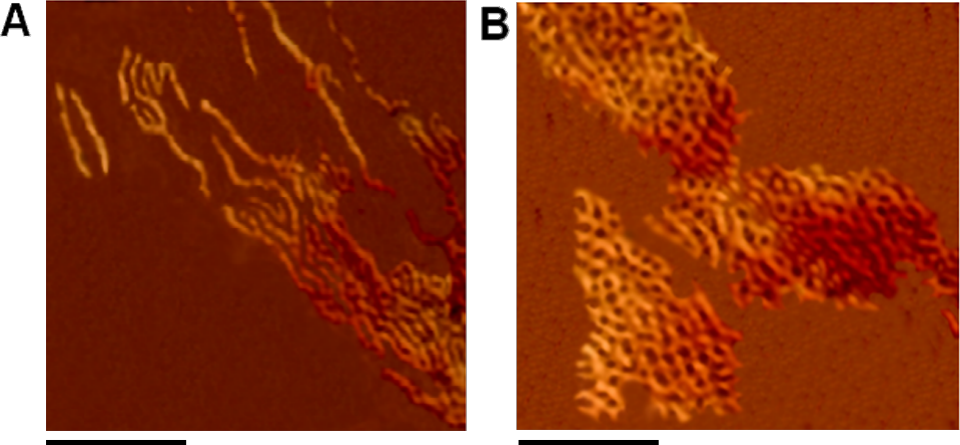
(A) Linear assembly of di-branched conjugates (R1)_2_–melamine and (R2)_2_–melamine. (B) Network assembly of tri-branched conjugates (R1)_3_–melamine and (R2)_3_–melamine. The black scale bar is 120 nm.

For direct visualization of the DNA–melamine hybrid assemblies, further investigation was done with hybridized solutions (2 nM) using high-resolution AFM (Figure 4). The di-branched DNA–melamine conjugates, (R1)_2_–melamine/(R2)_2_–melamine, were found to be aligned in linear streaks (Figure 4A). Many linear arrays are visible with lengths of ≈50 nm and 80–100 nm, which correlates well with the bands corresponding to the di-branched assembly in the native gel (Figure 3). The network array was observed from the self-assembly of tri-branched conjugates, (R1)_3_–melamine/(R2)_3_–melamine (Figure 4B). However, the network assembly was discontinuous at certain points, indicating hybridization defects. The average vertex length of the network was found to be approximately 9 ± 0.5 nm. This was expected since two melamine units are linked by a 24 base pair DNA of length ≈8.2 nm (24 × 0.34 nm = 8.16 nm, where 0.34 is the rise per base in a DNA double helix, Supporting Information File 1, Figure S4).

The circular dichroism (CD) spectra (see Supporting Information File 1, Figure S5) showed that the B-form of the duplex is maintained in the two self-assembled DNA–melamine nanostructures. However, a decline in the intensity of both the positive and negative bands for the linear and the network assemblies indicate the presence of conformational rigidity in those structures, which is more pronounced in the latter [35–36].

The electrostatic and non-covalent interactions between DNA and melamine have been previously reported [37–38]. Melamine was coupled to DNA by using disulfide chemistry to create DNA–dendron mono-conjugates [39]. We have elucidated a simple two-step aqueous phase phosphoramidation coupling reaction to prepare DNA–melamine–DNA building blocks in moderately good yields for DNA-based nanostructures. The resulting covalent conjugates of DNA and melamine are well-characterized by RP-HPLC, PAGE, MALDI–TOF and ESIMS. The purified di-branched and tri-branched conjugates were obtained after repeated reaction and PAGE purification. The self-assembly of the DNA–melamine–DNA building blocks was achieved by simple hybridization and further confirmed by PAGE, thermal melting studies, AFM and CD. What appears to be a disadvantage due to the formation of multiple products from the reaction was addressed by creating two distinct types of nanoassemblies. Fundamentally, this study offers further insight into the reaction of organic molecules with DNA, in particular, heterocyclic compounds that can offer attractive options to home nitrogen, sulfur or other interesting heteroatoms. Heterocyclic compounds could potentially be used to tune the molecular and electrical properties of a self-assembled system on the nanoscale. Moreover, being a zero linker approach with terminal phosphate as a reacting functionality, the methodology is inexpensive [27,40], does not require multiple synthetic steps and modifies the oligonucleotides with reactive functionalities. One noteworthy point about this EDC–imidazole-mediated cross coupling reaction is that restriction-digested fragments could be used to introduce an organic molecule of interest using this method. In addition, the resulting molecular building blocks are open to extension in both directions by relevant physico-chemical methods such as hybridization, polymerase chain reaction, and others [41–44]. The large increase in stability and melting temperature of the short duplexes could find a number of applications in biotechnology, such as in more sensitive DNA detection and diagnostics [30]. The self-assembled DNA–organic molecule hybrid forms a linear nanoassembly that could be used for various potential applications including templated polymerization or metallization.

Several strategies have been developed to generate self-assembled DNA nanostructures based on DNA–organic hybrid molecular building blocks. The selection of organic molecule and the DNA sequence are the most important considerations to direct the self-assembly of the hybrid structure. Researchers have independently achieved DNA assemblies in two and three dimensions by introducing organic molecules for various functions [45–47]. The use of such DNA–organic molecule hybrid structures and those reported here could be advantageous over the DNA-only based self-assembly process to create the nanoassembly. The presence of rigid organic or inorganic molecules at junctions of two or more DNA strands is a potential alternative to interweave DNA strands in a “DNA economic” way. Apart from structural rigidity and increased dynamic character, such structures can offer tunable electronic properties [30,48–49].

## Supporting Information

File 1 All experimental procedures and characterization of DNA–melamine conjugates and assemblies.
This file contains detail experimental methods, RP-HPLC chromatograms, ESI and MALDI mass spectrometry data and spectra, AFM images, and CD spectra.
