# Supplementary material for: DNA–melamine hybrid molecules: from self-assembly to nanostructures
Source: Beilstein J Nanotechnol. 2015 Jun 30;6:1432–8. doi: 10.3762/bjnano.6.148 (PMC4505151; doi:10.3762/bjnano.6.148)
Supplement: File 1 — All experimental procedures and characterization of DNA–melamine conjugates and assemblies. This file contains detail experimental methods, RP-HPLC chromatograms, ESI and MALDI mass spectrometry data and spectra, AFM images, and CD spectra. [file Beilstein_J_Nanotechnol-06-1432-s001.pdf]

Supporting Information

for

DNA–melamine hybrid molecules: from self-assembly to nanostructures

Rina Kumari[1], Shib Shankar Banerjee[2], Anil K. Bhowmick[3] and Prolay Das[1,* §]

Address: [1]Department of Chemistry, Indian Institute of Technology Patna, Patna 800013, India, [2]Department of Materials Science and Engineering, Indian Institute of Technology Patna, Patna 800013, India, and [3]Rubber Technology Centre, Indian Institute of Technology Kharagpur, Kharagpur 721302, India

Email: Prolay Das* - prolay@iitp.ac.in

* Corresponding author

[§]Tel: +91 612 255 2057, Fax: + 91 612 227 7383

All experimental procedures and characterization of DNA–melamine conjugates and assemblies

1) General: materials and methods

HPLC purified single-strand 24mer 5'-phosphorylated DNA were purchased from Sigma-Aldrich custom oligo. A solution of melamine was prepared by addition of a calculated amount of the compound in ultrapure water. All chemicals required for the buffer preparation and gel electrophoresis were obtained from either Sigma-Aldrich or Alfa Aesar and used without further purification.

2) Table S1: DNA strands used in the study.

R1	5'-PO4 TCG GAT AGT GCG GCT GTT GAC TGA 3' (24 base DNA, MW 7502.00)
R2	5'-PO4 TCA GTC AAC AGC CGC ACT ATC CGA 3' (24 base DNA, MW 7328.00)

3) Coupling of DNA with melamine

The coupling of 5'-phosphorylated ssDNA with melamine was carried out by adding 2 μL (1.0 nmol) aqueous solution of the single strand oligonucleotide to a solution of 0.5 mg of 1-ethyl-3-(3-dimethylaminopropyl)carbodiimide (EDC) and 10 μL of 0.1 M imidazole buffer solution at pH 6.0 with continuous stirring at 37 °C for 90 min. The resulting 5'-phosphorimidazolide ssDNA solution was purified by ethanol precipitation and redissolved in 10 μL of 0.1 M 4-(2-hydroxyethyl)-1-piperazineethanesulfonic acid

(HEPES-EDTA) buffer at pH 7.8. To this solution, 2 μL solution of melamine (0.2 nmol) was added and the reaction mixture was incubated at 55 °C with continuous stirring for 18 h. The crude reaction mixture was purified by dialysis using a 1000 Da MW membrane that eliminates the salts and unreacted melamine. Further purification was done by preparative denaturing PAGE.

4) Purification of DNA–melamine hybrid molecules by PAGE

DNA–melamine hybrid molecules were purified by preparative scale denaturing 30% PAGE to remove mono-conjugates and unreacted DNA. The desired bands were cut after EtBr staining and DNA was extracted with appropriate elution buffer followed by ethanol precipitation. In brief, the gel pieces were cut into very small pieces with a razor and incubated with 500 μL of elution buffer (0.5 M NH_4OAc 10 mm Mg $(OAc)_2$, 1.0 mM EDTA and 0.1% SDS) at 37 °C for 12 h. The gel pieces were removed by centrifugation at 14000 rpm at 0 °C for 30 min and the supernatant solution containing the DNA was collected and precipitated with ethanol. The conjugated products were air dried after ethanol washing and then redissolved in water and stored in an appropriate storage area for future use. The yield was calculated after obtaining the image of the gel and by comparison of the band intensity with the control DNA strands.

5) HPLC, MALDI-ToF and ESI-MS analysis of ssDNA–melamine conjugates

The ssDNA–melamine conjugates were analyzed by RP-HPLC (Shimadzu-VP, Kyoto, Japan) using a C18Q column (50 × 4.60 mm). Linear gradient elution was performed in 5–100% acetonitrile (CH_3CN) in 0.1 M triethylammonium acetate (TEAA) buffer (pH 7.0)

over 60 min at a flow rate of 1 mL/min with UV detection at 260 nm. The reaction mixture was diluted in 0.1 M TEAA buffer and 20 µL of the sample was injected during a single injection.

The covalent conjugates of DNA and melamine were purified from PAGE and subjected to molecular weight determination by Autoflex II MALDI-TOF-MS (Bruker Daltonics, Billerica, MA) using picolonic acid and dibasic sodium citrate as a matrix. Data processing was performed by the Flex Analysis Software. The mass of the conjugates were also confirmed by deconvoluted ESI-MS (Thermo).

6) Analytical HPLC profiles of ssDNA–melamine reaction mixture

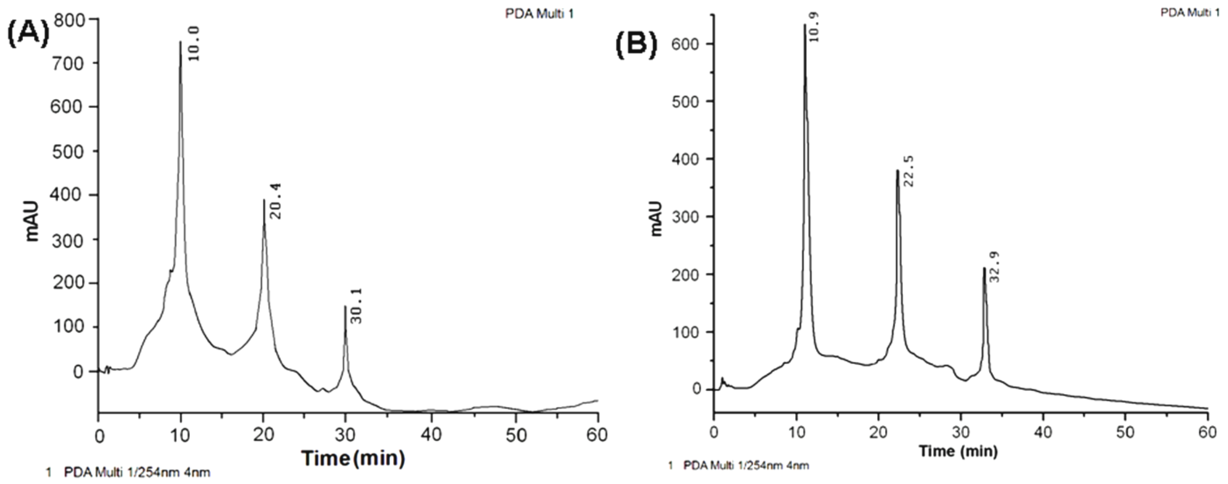

Figure S1: Analytical HPLC profiles of ssDNA–melamine reaction mixture (A) R1 DNA and melamine (B) R2 DNA and melamine.

7) Table S2: Theoretical and observed mass of di- and tri-conjugates of R1–melamine and R2–melamine.

Theoretical mass	Observed mass (MALDI-MS)	Observed mass (deconvoluted ESI-MS)	Conjugates
15095.7	15108.0	15090.0	R1–Melamine–R1
22580.5	22571.0	22577.0	$(R1)_3$–Melamine
14748.0	14750.7	14745	R2–Melamine–R2
22059.0	22038.2	22056	$(R2)_3$–Melamine

8) MALDI-ToF and ESI-MS spectra

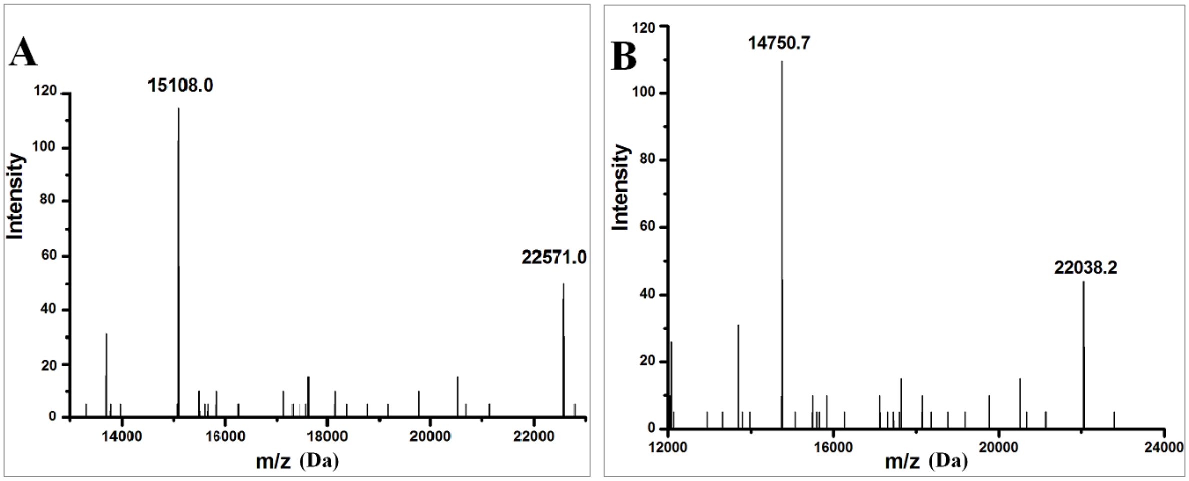

Figure S2: MALDI-ToF spectra of ssDNA–Melamine reaction mixture after purification. (A) R1–melamine conjugates. $(R1)_2$–melamine (m/z = 15108.0, theoretical m/z= 15095.7) and $(R1)_3$–melamine (m/z = 22571.0, theoretical m/z = 22580.52). (B) R2–

melamine conjugates. $(R2)_2$–melamine (m/z = 14750.7, theoretical m/z = 14748.0) and $(R2)_3$-melamine (m/z = 22038.2, theoretical m/z = 22059.0).

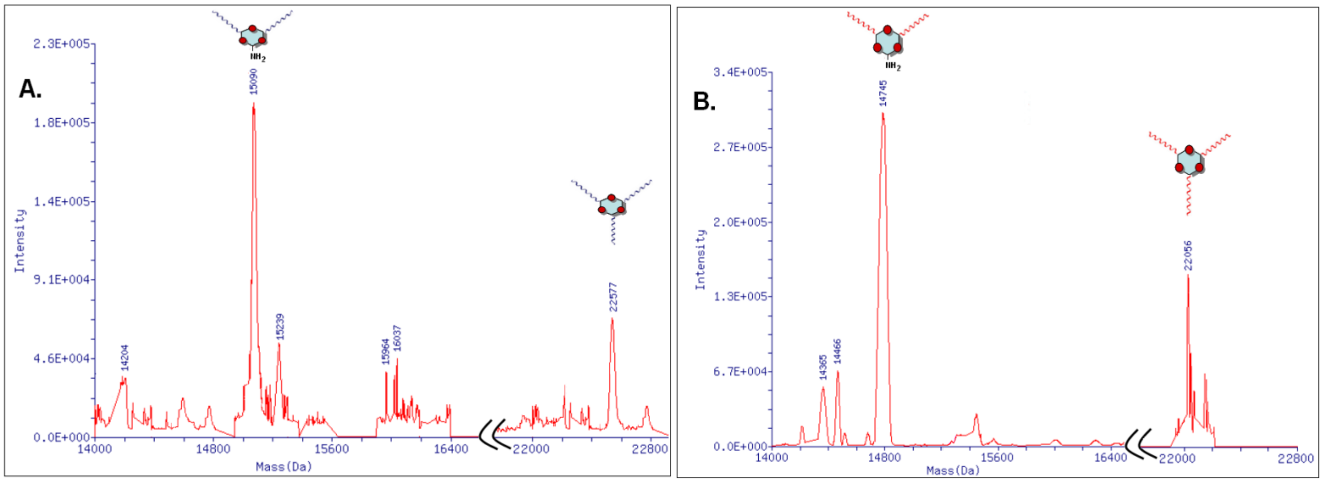

Figure S3: Deconvoluted ESI-MS DNA-Melamine conjugates (A) R1–melamine conjugates and (B) R2–melamine conjugates.

9) Thermal melting studies

The thermal melting of self-assembled products of tri-branched and di-branched DNA–melamine conjugates were studied separately by optical melting experiments using a Peltier-controlled UV–vis spectrophotometer (Bioquest, Cecil, U.K.). The equimolar amount (0.23 nmol of each) of two complementary ssDNA–melamine conjugates were annealed in sodium phosphate buffer (pH 7.0) in the presence of 10 mM $MgCl_2$ and 75 mM NaCl in a total volume of 60 µL by heating to 90 °C and allowing the solution to cool slowly to 4 °C at a rate of 1 °C per minute. This cycle was repeated four times for consistency of data. The temperature inside the cuvette was determined with a platinum probe. The absorbance data were analyzed by Origin 8.0 to obtain the melting

temperature of the samples. Similar experiments were also performed for control sample R1–R2 (normal oligomer duplex).

10) Native PAGE to detect self-assembly

The assemblies of DNA–melamine hybrid molecules were observed on native PAGE gel after hybridization. The equimolar amount (2.0 µM of each) of the two complementary tri-branched and di-branched ssDNA–melamine conjugates were hybridized separately in a total volume of 10 µL. Here, hybridization condition used was similar to thermal melting. The hybridized products were characterized by 10% non-denaturing PAGE run at 25 °C for 1 h at 200 V and stained with EtBr. The image was captured by a UVP-Gel Doc system.

11) Atomic force microscopy (AFM)

Samples were prepared by drop casting 20 µL of annealed DNA solution on freshly prepared APS mica. The aminopropylsilatrane (APS) was synthesized from triethanolamine and 3-aminopropyltriethoxysilane (APTES) in the laboratory. APS mica was prepared by treating the V1 quality mica sheets with synthesized APS following the procedure of Shlyakhtenko et al. [1]. Sample images were captured in intermittent contact mode atomic force microscopy (AC-AFM) using an Agilent 5500 scanning probe microscope. Commercial silicon nitride cantilevers with a force constant of 1.2–5.5 N/m were used for the measurements (MiKromash, Bulgaria). The cantilever was oscillating at its resonance frequency ranging from 60–90 kHz. The set point ratio of the cantilever,

which governs the tapping forces, varied from 0.2–0.4. The image was smoothed using Gaussian filtering.

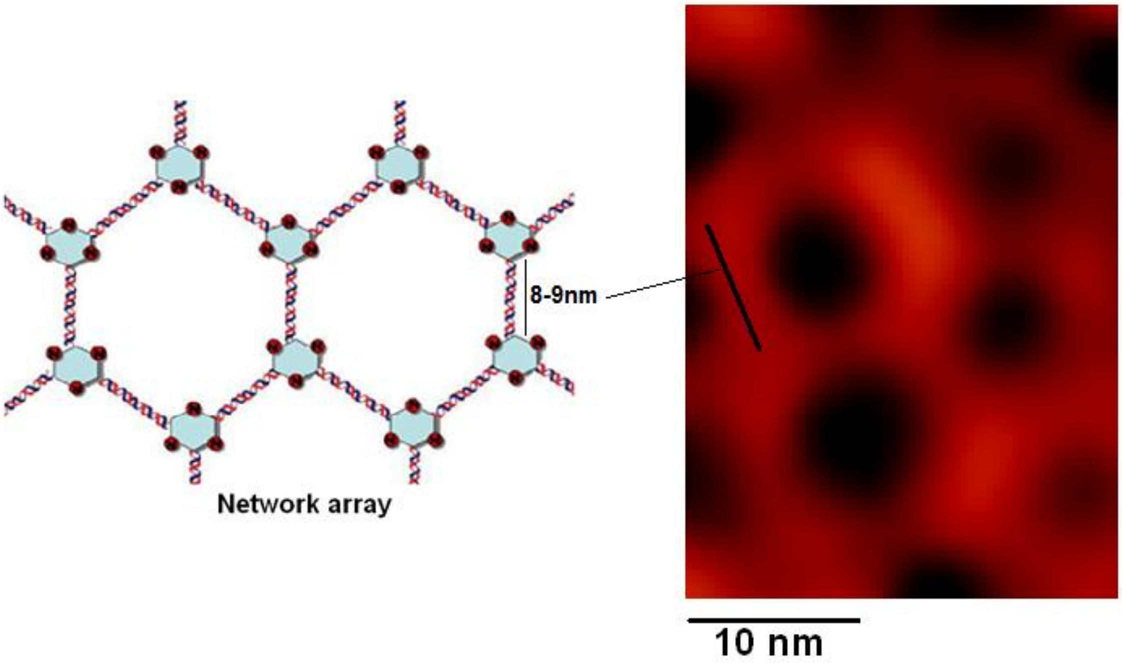

Figure S4: AFM image showing the average vertex length of the tri-branched assembly.

12) Circular dichroism (CD) studies

Circular dichroism (CD) spectra were obtained on a Jasco J-1500 spectropolarimeter with 200 µL samples containing hybridized R1–R2 and self-assembled melamine–DNA conjugates in 10 mM sodium phosphate buffer (pH 7.0) separately using a microcuvette with a 1 mm path length. The data were expressed as degrees of ellipticity (θ), in units of millidegrees (mdeg). Each sample was scanned three times from 220–450 nm with a scan rate of 50 nm/min.

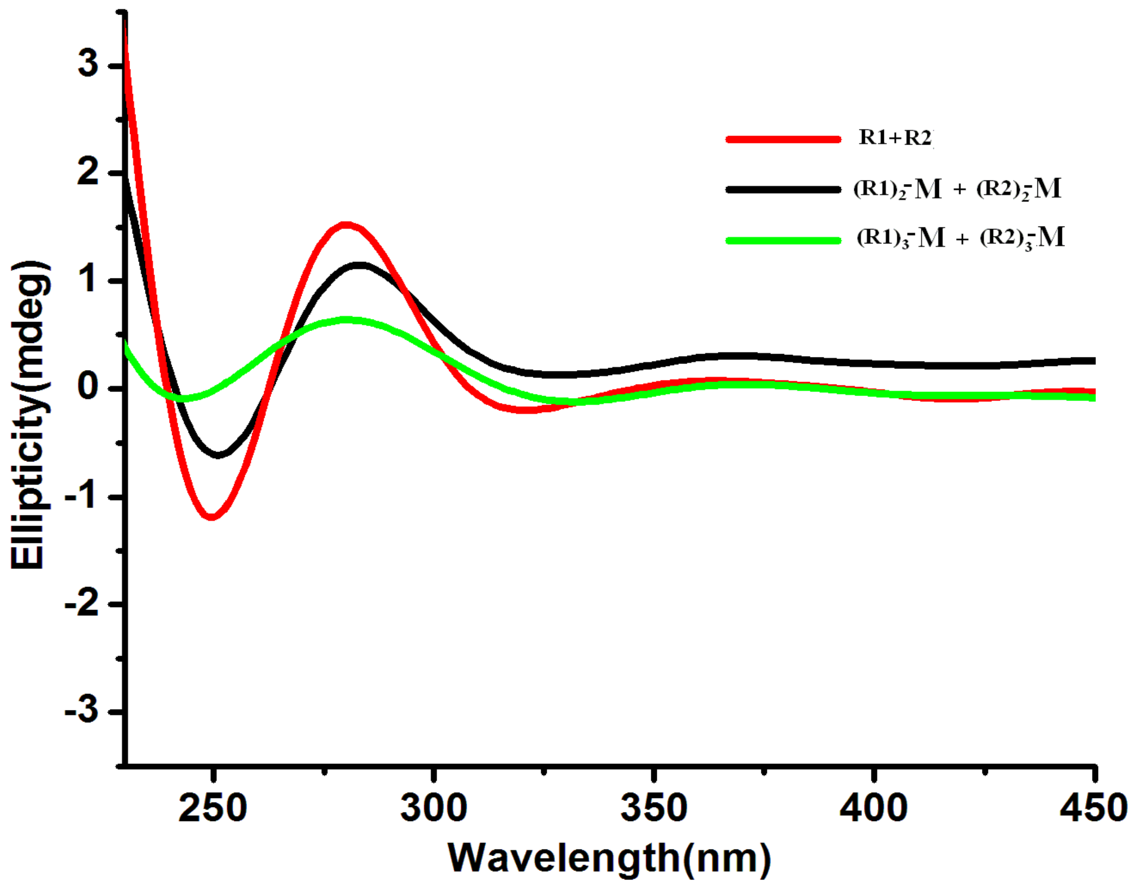

Figure S5: Circular dichroism spectra of the R1–R2 duplex DNA and self-assembled DNA–melamine conjugates.

References

[1] L. S. Shlyakhtenko, A. A. Gall, and Y. L. Lyubchenko, Methods Mol. Biol., 2013, 931, 295-312.
